# Distinct neural systems for men and women during emotional processing: a possible role of attention and evaluation

**DOI:** 10.3389/fnbeh.2012.00086

**Published:** 2012-12-10

**Authors:** Rashmi Gupta

**Affiliations:** Institute of Cognitive Neuroscience, University College LondonLondon, UK

**A commentary on**

**Men and women show distinct brain activations during imagery of sexual and emotional infidelity**

*by Takahashi, H., Matsuura, M., Yahata, N., Koeda, M., Suhara, T., and Okubo, Y. (2006). Neuroimage 32, 1299–1307*.

Among complex social emotions, there is a very important emotion called “romantic jealousy.” “Romantic jealousy is defined as a complex of thoughts, feelings, and actions which follow threats to self-esteem and/or threats to the existence or quality of the relationship, when those threats are generated by the perception of a real or potential attraction between one's partner and a (perhaps imaginary) rival” (White, 1981, p. 24). Takahashi et al. (2006) investigated neural correlates associated with jealousy and found that men and women have different neuropsychological modules to process sexual and emotional infidelity. Given the paucity of studies in the literature, their finding helps to understand sex-specific biological underpinnings of romantic jealousy and broadens the knowledge of this field.

Takahashi et al. (2006) discussed possible distinct neural systems for men and women during sexual and emotion infidelity. They explained their results in terms of arousal associated with emotional information, hormonal transmission, and evolutionary perspective. However, they did not discuss a possible role of “*attention*” or “*attentional networks*”; and “attention and evaluative bias” to explain their results, which is the main focus of this paper.

Takahashi et al. (2006) used functional magnetic resonance imaging, during imagination of sexual and emotional infidelity conditions, to investigate the difference in neural substrates between males and females. Participants were provided with 18 short sentences for each category (neutral, sexual infidelity, and emotional infidelity). They were required to read the sentences silently and were told to imagine the situation described in sentences. After the scan, participants rated the sentence according to how they would feel if the scenario protagonist was their girlfriend/boyfriend. They rated the intensity of jealousy and other basic emotions (anger, sadness, surprise, fear, disgust, and happiness) for each sentence.

The full brain volumes were imaged using 40 transaxial contiguous with a slice thickness of 3 mm. To assess the specific condition effect, they used the contrasts of sexual infidelity minus neutral (SI-N) and emotional infidelity minus neutral (EI-N). Correlation coefficients between the degree of activation and rating of jealousy for emotional infidelity were also calculated.

They did not find gender differences in ratings, but they did find neural differences. Common brain areas such as the frontal regions (medial frontal gyrus and middle frontal gyrus) and the cingulate cortex were activated in both groups for the emotional infidelity condition. However, men demonstrated greater activation in the amygdala and hypothalamus than women. In contrast, women demonstrated greater activation in the posterior superior temporal sulcus (pSTS) (angular gyrus). A positive linear correlation was also observed between self-rating of jealousy for emotional infidelity and the degree of activation in insula for men and in pSTS for women. Takahashi et al. (2006) explained their results in terms of arousal associated with emotional information, hormonal transmission, and evolutionary perspective.

However, in my opinion, they did not discuss a possible role of “*attention and evaluative bias*” toward the processing of negative information, which might contribute gender differences in the processing of emotional information. It has been reported that females showed remarkable attention and evaluative bias even for the processing of moderately negative information, whereas males showed neither biases for these information (Wrase et al., 2003; Hofer et al., 2006; Li et al., 2008). Li et al. (2008) found gender difference in N2 and P3b ERP components, and are involved in early attention and later evaluative processes respectively, which are influenced by emotional saliency. Enhanced activity observed in N2 and P3b component in females during the processing of less negative information suggests that females are more susceptible for attention and evaluative bias for processing of negative information. This may make them more sensitive to think and respond more intensely to issues, signifying social rejection factor in emotional infidelity than men. It could result in more activation in pSTS, in females than men observed in Takahashi et al. (2006) study, which are critically involved in social interaction.

Apart from gender differences, Takahashi et al. (2006) did not discuss a possible role of “*attention*” or “*attentional networks*” in the processing of emotional information, specifically in the emotional infidelity condition. It should be noted that in the emotional infidelity condition both groups activated common brain areas such as frontal regions (medial frontal gyrus and middle frontal gyrus) and the cingulate cortex, both of which areas are involved in attentional networks. Pessoa et al. (2002) suggested that the attentional networks also involve fronto-parietal regions, including the middle frontal gyrus, anterior cingulate cortex, inferior frontal gyrus, and anterior insula. These brain areas are also recruited in the processing of emotional information. Pessoa et al. (2002) suggested that emotional processing and attentional processing activate similar brain regions and the overlapping brain networks of emotion and attentional processing suggests a role of attention in emotion processing. This explanation fits with the neuroimaging finding that neural processing of emotion requires attention (Pessoa, 2009). This neuroanatomical link between attention and emotion processing was not made explicit by Takahashi et al. (2006), despite the important implications it has for the role of attentional mechanisms serving emotional processing.

Together with explanations reported in the Takahashi et al. (2006) study, it is also very important to consider “*attention and evaluative biases*,” and “*attentional network*” mechanisms underlying emotional processing in males and females. These interpretations have implications to understanding the complete picture of gender differences observed in neural processing of emotion information. Future work should be done toward investigating the functional differences between males and females for different emotion processing.
